# Exercise preconditioning improves electrocardiographic signs of myocardial ischemic/hypoxic injury and malignant arrhythmias occurring after exhaustive exercise in rats

**DOI:** 10.1038/s41598-022-23466-5

**Published:** 2022-11-05

**Authors:** Yuan-Pan Guo, Shan-Shan Pan

**Affiliations:** School of Kinesiology, Shanghai University of Sport, 399 Changhai Road, Shanghai, 200438 China

**Keywords:** Biochemistry, Molecular biology, Physiology

## Abstract

Exercise preconditioning (EP) has a good myocardial protective effect. This study explored whether EP improves electrocardiographic (ECG) signs of myocardial ischemic/hypoxic injury and the occurrence of malignant arrhythmia after exhaustive exercise. A total of 120 male SD rats were randomly divided into the control group (group C), early exercise preconditioning group (group EEP), late exercise preconditioning group (group LEP), exhaustive exercise group (group EE), early exercise preconditioning + exhaustive exercise group (group EEP + EE) and late exercise preconditioning + exhaustive exercise group (group LEP + EE). Changes in heart rate (HR), ST segment, T wave and QT corrected (QTc) intervals on ECG; hematoxylin-basic fuchsin-picric acid (HBFP) staining; and cTnI levels were used to study myocardial injury and the protective effect of EP. Compared with those in group C, the levels of plasma markers of myocardial injury, HBFP staining and ECG in group EE were significantly increased (*P* < 0.05). Compared with those in group EE, the levels of plasma markers of myocardial injury, HBFP staining and ECG in group EEP + EE and group LEP + EE were significantly decreased (*P* < 0.05). The results suggested that EP improved ECG signs of myocardial ischemic/hypoxic injury and malignant arrhythmias that occur after exhaustive exercise. The ST segment and T wave could also serve as indexes for evaluating exhaustive exercise-induced myocardial ischemia/hypoxia.

## Introduction

Exercise is an effective lifestyle intervention to reduce the risk factors for cardiovascular disease and cardiac events^1^. A form of acute cardiac protection can occur after exercise^2^. Exercise preconditioning (EP) is established by a bout of repeated high-intensity intermittent aerobic exercise that can activate an endogenous protective effect on the heart and protect myocardial tissues against subsequent persistent ischemia/hypoxia injury^3–5^. EP can be divided into early exercise preconditioning (EEP) and late exercise preconditioning (LEP). EEP occurs immediately after short-term, high-intensity intermittent aerobic exercise and lasts for 1–3 h^3,6^; LEP occurs 24 h after short-term, high-intensity intermittent aerobic exercise and can last for several days^2,3^. EP can improve myocardial ischemia/hypoxia, reduce the area of myocardial infarction^3,5,7,8^, prevent myocardial stunning^9^, and alleviate exhaustive exercise-induced myocardial injury^10^.

The protective effect of EP against exhaustive exercise-induced myocardial injury has been confirmed. To demonstrate the myocardial protective effect of EP, Sun et al.^11^ used plasma markers of myocardial injury combined with histological staining to verify exhaustive exercise-induced myocardial ischemic/hypoxic injury in rats. Cardiac troponin I (cTnI) is blood markers of myocardial injury. In an animal study^12^, the authors measured the levels of cTnI in rat cardiac plasma and performed myocardial ischemia/hypoxia staining, finding a protective effect of EP against exhaustive exercise-induced myocardial ischemic/hypoxic injury. Other animal studies^4,13^ have also involved the use of plasma markers of myocardial injury and myocardial ischemia/hypoxia staining to explore the myocardial protective effect of EP. Electrocardiogram (ECG) signals can provide information on the rhythm, structure and function of the heart; the features of a typical normal ECG waveform include the P wave, QRS complex, T wave, RR interval, PR interval, QT interval and lengths of the PR and ST segments^14^. Among these features, the ST segment and T wave are indicators of myocardial ischemia/hypoxia on ECG^15^. A study showed that isoproterenol injection induced acute myocardial ischemic/hypoxic injury, which caused significant ST segment elevation and QT prolongation on ECG in rats^16^. Exhaustive exercise can also induce myocardial ischemic/hypoxic injury, significantly increasing arrhythmia and ST segment depression in healthy middle-aged men^17^. An animal model study showed that long-term intensive exercise training could increase arrhythmia inducibility^18^. However, a reasonable and appropriate exercise regimen can reduce human myocardial ischemia/hypoxia caused by coronary occlusion and significantly reduce ST segment elevation^19^. Heart rate (HR) is a phase indicator of cardiac function. The increase in HR during exercise and subsequent recovery afterwards have often been associated with all-cause and cardiovascular mortality^20^, and EP was found to be effective in reducing HR after exhaustive exercise^21^.

Hematoxylin-basic fuchsin-picric acid (HBFP) staining is an ischemia/hypoxia stain that can show high-intensity exercise-induced myocardial ischemic/hypoxic injury, with injured cardiomyocytes being stained scarlet^4,22^. ST segment and T wave reflect myocardial ischemia/hypoxia from the perspective of electrophysiology, while HBFP staining reflects the degree of myocardial ischemia/hypoxia from a histological perspective. Among others, our main hypotheses were as follows: (1) EP can improve the electrocardiographic characteristics of myocardial ischemia/hypoxia induced by exhaustive exercise and reduce the occurrence of exhaustive exercise-induced malignant arrhythmia; and (2) the changes in the ST segment and T wave of ECG are strongly correlated with the results of HBFP staining of the myocardium.

This study focused on the changes in HR, ST segment, T wave and QT corrected (QTc) interval on ECG; HBFP staining; and levels of the plasma marker of myocardial injury, cardiac troponin I (cTnI), to study myocardial injury and the protective effect of EP. We explored the electrocardiographic characteristics of exhaustive exercise-induced myocardial ischemic/hypoxic injury and the changes in malignant arrhythmia that occur after exhaustive exercise and EP, and we addressed the correlation of the ST segment and T wave with the ischemic/hypoxic area as indicated by HBFP staining. This study adds to the available methods for evaluating the myocardial protective effect of EP and provides a theoretical basis for follow-up research.

## Materials and methods

### Animals

A total of 120 healthy adult male Sprague–Dawley (SD) rats weighing 203 ± 7 g (Jiesijie Laboratory Animal Co. Ltd., Shanghai, China) were used. They were housed under standard housing conditions with a 12/12-h light/dark cycle at a temperature of 22–24 °C and a humidity level of 40–70%, and they were allowed to eat freely. All animal experiments were performed according to the Laboratory Animal Guideline for Ethical Review of Animal Welfare (GB/T 35892-2018, China) and approved by the Ethics Committee of Science Research at Shanghai University of Sport, China (2015001).

### Experimental protocol

**Figure 1:**
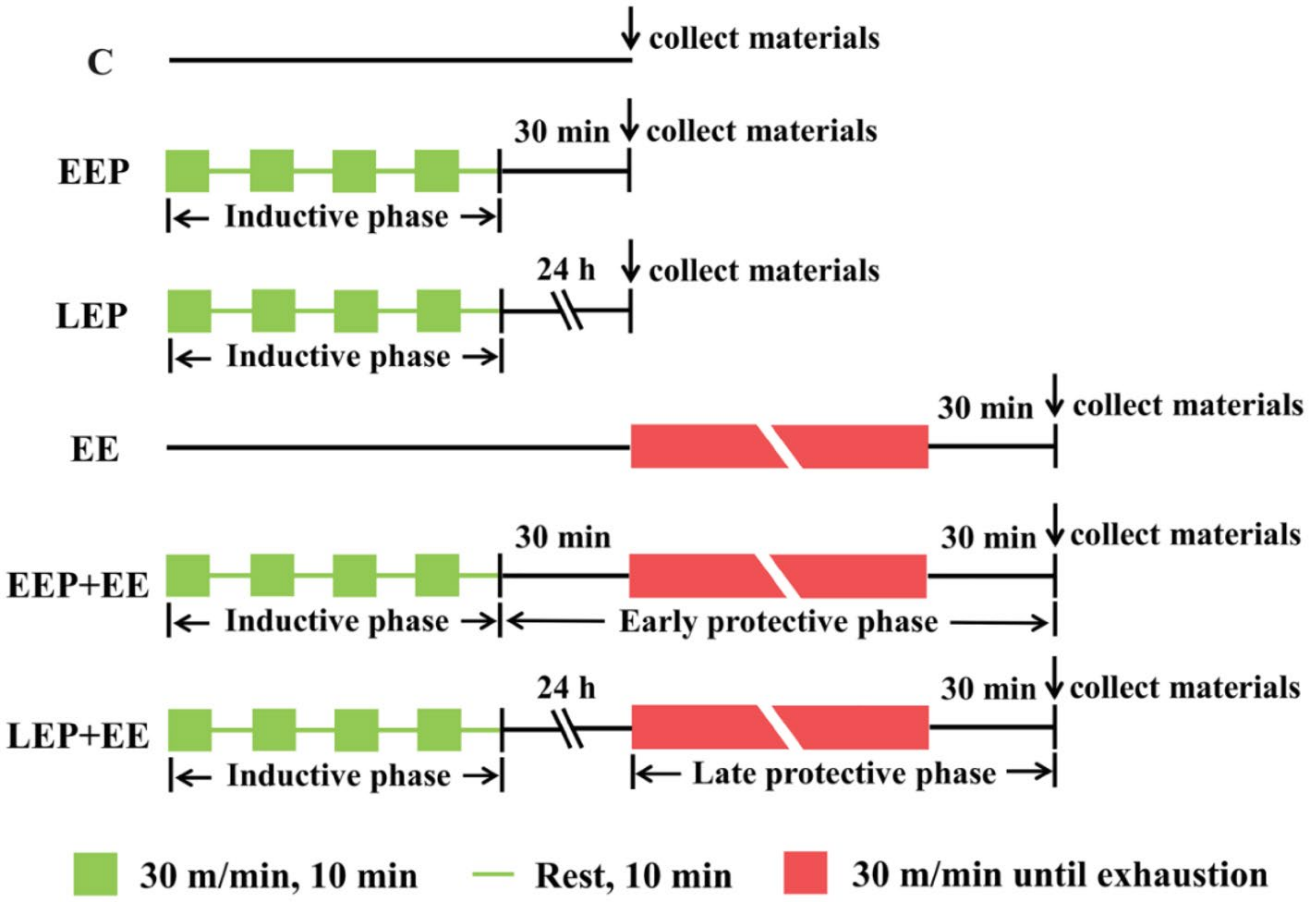
Experimental protocol^24^. C, control group; EEP, early exercise preconditioning group; LEP, late exercise preconditioning group; EE, exhaustive exercise group; EEP + EE, early exercise preconditioning plus exhaustive exercise group; LEP + EE, late exercise preconditioning plus exhaustive exercise group.

The experimental protocol in Fig. 1 was based on previous studies^3,4^. All animals were fed adaptively for a week, during which adaptive treadmill training at 15 m/min and a 0% grade was carried out 10 min each day for 5 days, and the animals rested for 1 day after adaptive training. Then, they were randomly divided into 6 groups. In the control group (group C), rats were placed on the treadmill without substantial exercise. In the early exercise preconditioning group (group EEP), rats ran on the treadmill at 30 m/min for 10 min and then rested for 10 min to complete the cycle; 4 such cycles were conducted to establish the EP model (approximately 75% VO_2_max^23^). The rats were anesthetized, and ECG was recorded after the completion of EP. Then, samples were harvested 30 min after EP. In the late exercise preconditioning group (group LEP), rats were run on a treadmill to establish the EP model, and ECG and sample harvesting were performed 24 h after EP. In the exhaustive exercise group (group EE), rats were run on a treadmill at 30 m/min until exhaustion to establish a model of exhaustive exercise-induced myocardial injury in rats. The standard indicator of exhaustion from running in rats is the loss of the righting reflex; the rats are unable to right themselves when placed on their backs^24^. In the early exercise preconditioning + exhaustive exercise group (group EEP + EE), rats performed EP, rested for 30 min, and then ran on a treadmill at 30 m/min until exhaustion. In the late exercise preconditioning + exhaustive exercise group (group LEP + EE), rats performed EP, rested for 24 h, and then ran on a treadmill at 30 m/min until exhaustion. ECG was recorded in group EE, group EEP + EE and group LEP + EE after the completion of exhaustive exercise, and samples were harvested 30 min after the exhaustive exercise.

### Recording and analysis of electrocardiograms

ECG signals of the rats were acquired with an animal ECG acquisition and analysis system (SP2006, Beijing Softlong Biotechnology Co., Ltd., Beijing, China). After intraperitoneal anesthesia with 0.4% (1 ml/100 g) pentobarbital sodium, the animals were positioned with the ventral side up. The limbs of the rats were fixed on the anatomical table with adhesive tape; the limb leads of the animal ECG were connected to the limbs of the rats, and then the ECG acquisition system was turned on to record the ECG signals of rats. ECG data were collected from each rat for 3 min. In this study, the ST segment and T wave were used to evaluate myocardial ischemic/hypoxic injury. The QTc interval was used to evaluate the occurrence of malignant arrhythmia induced by exhaustive exercise. HR was obtained from the ECG.

### Tissue harvesting

After the ECG signals of the rats were collected, the abdominal cavity was opened to collect 5 ml of blood from the posterior vena cava, and then the chest was opened to expose the heart. A perfusion needle was inserted at the apex of the heart; 1% heparin was injected, 0.85% saline was infused, and the posterior vena cava was cut quickly. Then, 4% paraformaldehyde solution was used for perfusion fixation. After perfusion was finished, the heart was removed and fixed for 24 h in 4% paraformaldehyde. Then, the heart was routinely dehydrated, cleared and embedded in paraffin, and myocardial sections were prepared for ischemia/hypoxia staining.

### Determination of plasma cTnI level

A chemiluminescent immunoassay (CLIA) was used to measure the plasma cTnI level (assay kit: Beckman, A98264, USA). Blood samples were centrifuged for 15 min at 3000 rpm to separate the plasma. The quantification is based on a two-site enzyme immunoassay. The solid phase is created by paramagnetically coating a surface with an anti-cTnI monoclonal antibody. An anti-cTnI monoclonal antibody conjugated with alkaline phosphatase then binds to the cTnI antigens captured by the fixed anti-cTnI antibodies in situ. The level of the substance to be measured in the sample was quantified according to the stored multipoint calibration curve, and the sensitivity range for the measurement of plasma cTnI level was 0.02–100 ng/ml.

### Imaging and analysis of HBFP staining

Slides were soaked in hematoxylin solution for 5 min, washed with distilled water to remove the excess stain, and then soaked in 1% hydrochloric acid-alcohol for 5 s for differentiation. Next, the slide was immersed in 0.1% basic fuchsin (Sangon Biotech, A502770-0025, China) solution for 40 s and finally dipped in 0.1% picric acid-acetone solution for 15–20 s. The slides were cleared with xylene and fixed with neutral balsam (SCR, 10004160, China). An Olympus microscope (BX53, Japan) and Olympus microscopic digital camera (DP80, Japan) were used to observe the results and acquire images (we chose a × 10 eyepiece and × 40 objective for observation). Five stained sections were randomly selected from each group, and five fields (× 400) were randomly selected from each section. The images were analyzed with Image-Pro Plus 6.0 software (Media Cybernetics, Silver Spring, MD, USA). The image to be analyzed was uploaded to the software, and “Select Colors” was chosen. We then clicked “Color Sucker” to select the scarlet area in the HBFP-stained image. Next, in the “Count/Size” interface, we clicked “Count-Measure-Select Measurements” and selected the parameters “Area” and “IOD”. We next clicked “View-Statistics” to count the parameters and obtain the data. The extent of myocardial ischemic/hypoxic injury was shown with the ischemia/hypoxia area (IHA), the degree of myocardial ischemic/hypoxic injury was shown with the integral optical density (IOD), and the degree of ischemic/hypoxic injury per unit area of myocardium was shown with the mean optical density (MOD, where MOD = IOD/IHA). In this study, the MOD was used to evaluate exercise-induced myocardial injury and protection.

### Statistical analysis

The data from the experiment were analyzed by Statistical Product and Service Solutions 20.0 (SPSS 20.0; IBM, USA). Normally distributed results are expressed as the mean ± SD and were analyzed by one‑way analysis of variance (ANOVA) followed by Fisher’s least significant difference (LSD) test. Nonnormally distributed results are presented as the median (IQR), and the Kruskal–Wallis (K-W) test was used for analysis. Correlation analysis between the ST segment, T wave and HBFP staining results in rats was based on the bivariate correlation. Differences were considered statistically significant at *P* < 0.05.

### Ethics approval

We designed the experimental protocol (including the research questions, key design characteristics, and analysis plan) before the study, which was in accordance with the ARRIVE guidelines.

## Results

### Collection and analysis of electrocardiograms

#### Heart rate

**Figure 2:**
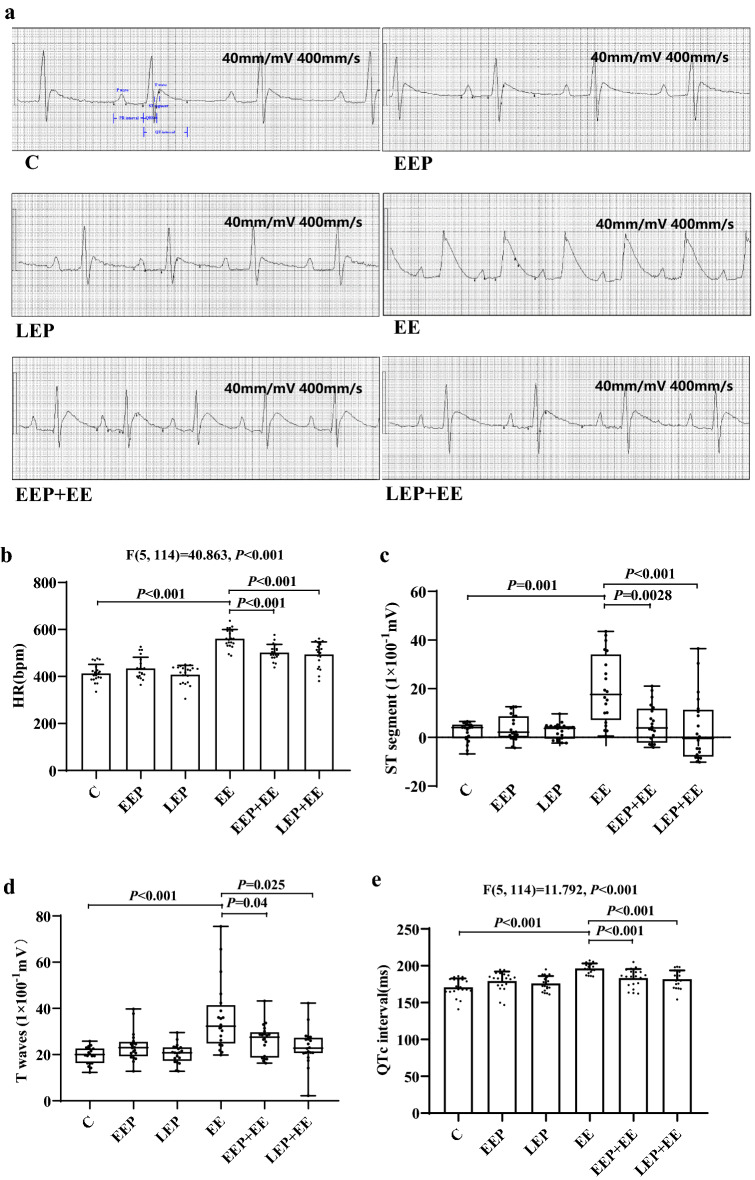
ECG analysis results in rats. (**a**) The ECG waves in rats (40 mm/mV, 400 mm/s). (**b**) HR results in each group of rats (LSD test). (**c**) Changes in ST segments on ECG (K-W test). (**d**) Changes in T waves on ECG (K-W test). (**e**) Changes in QTc intervals on ECG (LSD test). The elevation of the ST segment in group EE was significantly higher than that in group C, and the ST segments of group EEP + EE and group LEP + EE were significantly lower than that of group EE (**a**,**c**). The T wave of group EE was significantly higher than that of group C; the T waves were significantly lower in group EEP + EE and group LEP + EE than in group EE (**a**,**d**). The QTc interval in group EE was obviously prolonged compared with that in group C, and the QTc intervals in group EEP + EE and group LEP + EE were obviously shortened compared with that in group EE (**a**,**e**). Compared with that of rats in group C, the HR of rats in group EE was significantly higher. Compared with that of rats in group EE, the HR of rats in group EEP + EE and group LEP + EE was significantly lower. The results suggested that EP could significantly decreased the HR during exhaustive exercise (**b**). The results suggested that EP could effectively reduce the extent of ischemia/hypoxia induced by exhaustive exercise and that the occurrence of malignant arrhythmia, which was increased by exhaustive exercise, could be improved by EP.

The HR results are shown in Fig. 2b. Compared with that in group C, the HR in group EE was significantly elevated (*P* < 0.001). Compared with that in group EE, the HR in group EEP + EE (*P* < 0.001) and group LEP + EE (*P* < 0.001) was significantly reduced.

#### ST segment and T wave

The waves of the ECG signal from the rats are shown in Fig. 2a; the ST segment and T wave on the ECG in group C were normal, and there was no obvious change in the ST segment or T wave of group EEP or group LEP; however, both the ST segment and T wave were obviously elevated in group EE. In group EEP + EE and group LEP + EE, the ST segment was elevated, and the T wave was slightly elevated.

The results of the ST segments and T waves on ECG from the rats are shown in Fig. 2c,d; compared with group C, group EE exhibited significantly higher ST segment elevation (*P* = 0.001) and T waves (*P* < 0.001). Moreover, the ST segments in group EEP + EE (*P* = 0.028) and group LEP + EE (*P* < 0.001) and the T waves in group EEP + EE (*P* = 0.04) and group LEP + EE (*P* = 0.025) were significantly reduced compared with those in group EE.

#### QTc interval

The results of the QTc intervals on ECG from the rats are shown in Fig. 2e. Compared with those in group C, the QTc intervals in group EE were obviously increased (*P* < 0.001). Compared with those in group EE, the QTc intervals in group EEP + EE (*P* < 0.001) and group LEP + EE (*P* < 0.001) were obviously decreased.

### Plasma cTnI level quantification

**Figure 3:**
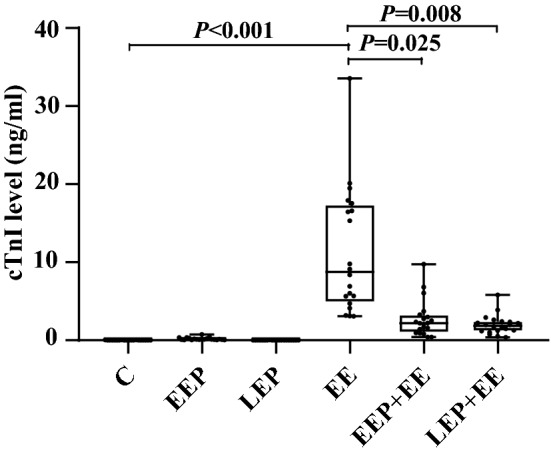
Results of plasma cTnI measurement (K-W test)**.** Compared with that in group C, the plasma cTnI level was significantly higher in group EE. Compared with that in group EE, the plasma cTnI level was significantly lower in group EEP + EE and group LEP + EE. The results suggested that exhaustive exercise induced myocardial injury and that EP had a protective effect against exhaustive exercise-induced myocardial injury.

The results of plasma cTnI measurement are shown in Fig. 3. Compared with those in group C, plasma cTnI levels were significantly elevated in group EE (*P* < 0.001). Compared with those in group EE, plasma cTnI levels in group EEP + EE (*P* = 0.025) and group LEP + EE (*P* = 0.008) were significantly reduced.

### Analysis of HBFP staining images

**Figure 4:**
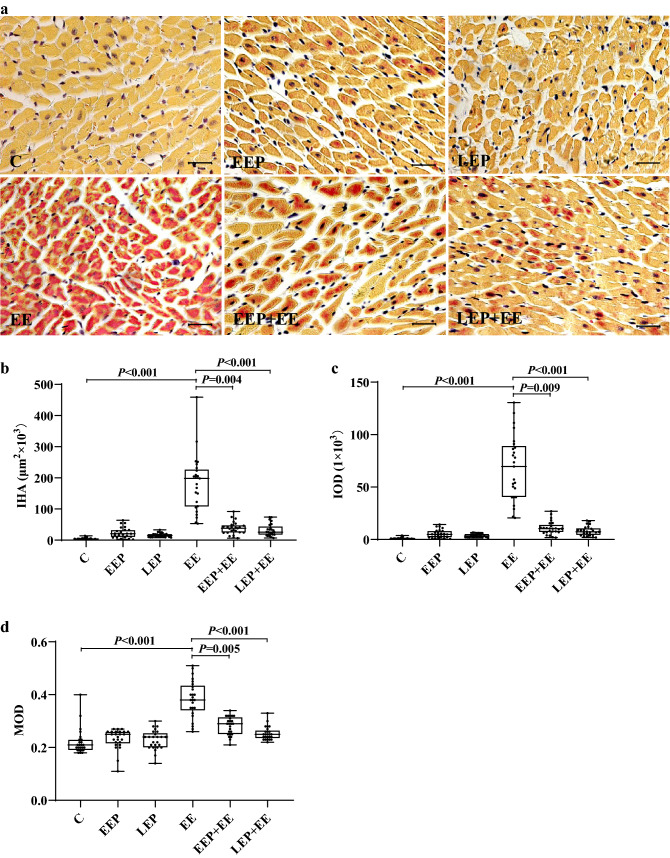
Analysis of the myocardium in HBFP staining images. (**a**) The results of HBFP staining of the myocardium (scale bar: 20 μm). There were a large number of scarlet-stained cardiomyocytes in group EE. The number of scarlet-stained cardiomyocytes was obviously reduced in the EEP + EE and LEP + EE groups. (**b**) The analysis of IHA in HBFP staining images (K-W test). (**c**) The IOD of HBFP staining images (K-W test). (**d**) The MOD of HBFP staining images (K-W test). The IHA, IOD, and MOD were significantly elevated in group EE. Compared with those in group EE, the IHA, IOD, and MOD in group EEP + EE and group LEP + EE were significantly lower. The results suggested that exhaustive exercise induces severe ischemic/hypoxic injury in the myocardium and that EP can effectively mitigate the ischemic/hypoxic injury caused by exhaustive exercise.

The results of HBFP staining of the myocardium are shown in Fig. 4a. Normal cardiomyocytes were stained yellow, and ischemia/hypoxia-injured cardiomyocytes were stained scarlet by HBFP staining. Cardiomyocytes in group C had clear boundaries, and some blue–violet nuclei were surrounded by yellow-stained cytoplasm. There were some ischemic/hypoxic changes in the cardiomyocytes in group EEP and group LEP, and a few scarlet cardiomyocytes were scattered in the myocardium. In group EE, there were many scarlet-stained cardiomyocytes that were obvious ischemic/hypoxic changes compared with those in group C. Compared with those in group EE, the number of scarlet-stained cardiomyocytes was obviously reduced, and the degree of ischemia/hypoxia in cardiomyocytes was significantly improved, in group EEP + EE and especially in group LEP + EE.

The IHA results of the HBFP staining image analysis are shown in Fig. 4b. Compared with that of group C, the IHA of group EE (*P* < 0.001) was significantly higher; compared with that of group EE, the IHAs of group EEP + EE (*P* = 0.004) and group LEP + EE (*P* < 0.001) were significantly reduced. The IOD results of the HBFP staining image analysis are shown in Fig. 4c. Compared with that of group C, the IOD of group EE (*P* < 0.001) was significantly elevated; compared with that of group EE, the IODs of EEP + EE (*P* = 0.009) and group LEP + EE (*P* < 0.001) were significantly reduced. The MOD results of the HBFP staining image analysis are shown in Fig. 4d. Compared with that of group C, the MOD of group EE (*P* < 0.001) was significantly elevated. Compared with that of group EE, the MODs of group EEP + EE (*P* = 0.005) and group LEP + EE (*P* < 0.001) were significantly reduced.

### Correlation of ST segments and T waves with HBFP staining

**Figure 5:**
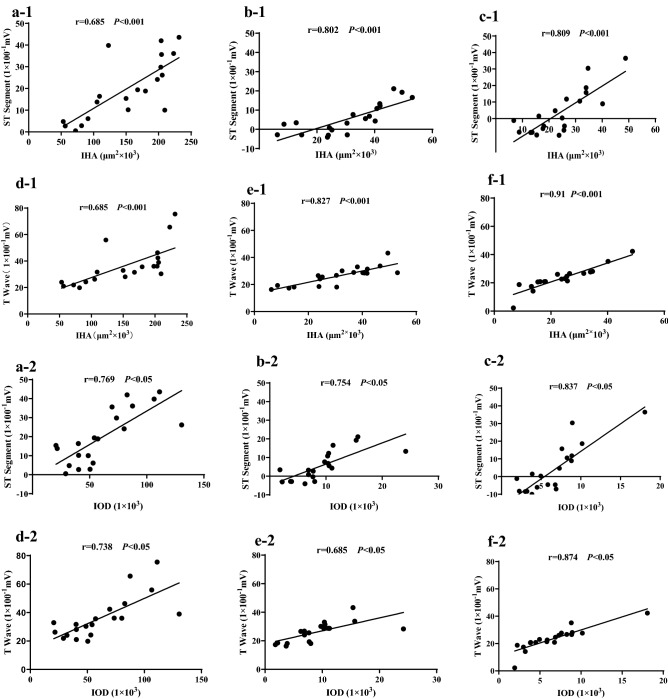
Correlation analysis of ST segments and T waves with HBFP staining. The results of correlation analysis of ST segments (**a**–**c**) and T waves (**d**–**f**) with HBFP staining in group EE (**a**,**d**), group EEP + EE (**b**,**e**) and group LEP + EE (**c**,**f**). The results suggested that ST segments and T waves on ECG had a strong positive correlation with HBFP staining.

Exhaustive exercise significantly changed the ST segments and T waves of the ECG signal in the rats. The IHA on HBFP staining images shows the extent of myocardial ischemic/hypoxic injury, and the IOD shows the degree of myocardial ischemic/hypoxic injury. Therefore, we analyzed the correlation of the ST segments and T waves on ECG with the IHA and IOD on HBFP staining images in group EE, group EEP + EE and group LEP + EE. The results of correlation analysis of ST segments and T waves with HBFP staining in Fig. 5. The results showed that ST segments with HBFP staining in group EE (Fig. 5a-1,a-2), group EEP + EE (Fig. 5b-1,b-2) and group LEP + EE (Fig. 5c-1,c-2) were positive correlation obviously (*P* < 0.001). The results showed that T waves with HBFP staining in group EE (Fig. 5d-1,d-2), group EEP + EE (Fig. 5e-1,e-2) and group LEP + EE (Fig. 5f-1,f-2) were positive correlation obviously (*P* < 0.001).

## Discussion

### EP reduces the high heart rate caused by exhaustive exercise

HR, an index that reflects the state of cardiovascular function, is obviously influenced by exercise and is an important basis for determining exercise intensity^25,26^. A study showed that short-term exercise training could enhance the inotropic response to beta-adrenergic stimulation and that this enhancement was associated with increases in cardiac output and stroke volume during peak exercise^27^. The increase in HR during exercise is, for the most part, attributable to the decrease in vagal tone followed by increases in sympathetic output and levels of circulating catecholamines^28^. Due to the effect of heterometric autoregulation, when the HR increases to a certain extent, the cardiac output decreases^29^. Oxygen demand increases significantly in exhaustive exercise^30^. The decreases in cardiac output during exhaustive exercise will likely lead to myocardial ischemia/hypoxia and cause injury^31^.

A study^21^ found HR was significantly increased in rats after exhaustive exercise, and EP can effectively reduce exhaustive exercise-induced high HR. In this study, The HR of rats increased significantly after exhaustive exercise, but the HRs of rats in group EEP + EE and group LEP + EE were significantly decreased compared with those of rats in group EE, the results are in line with Su et al.^21^’ s findings. These results showed that short-term, high-intensity interval aerobic exercise had a good protective effect against injury caused by subsequent exhaustive exercise and reduced the HR of rat after exhaustive exercise. A previous study demonstrated a markedly attenuated chronotropic response to beta1-adrenergic stimulation in endurance athletes with a low HR compared with that of sedentary subjects^32^. Exercise regulates the activity of the autonomic nervous system and enhances cardiac parasympathetic regulation, resulting in bradycardia^33,34^, which perhaps explains why EP is effective in reducing HR after exhaustive exercise. Exhaustive exercise-induced myocardial ischemic/hypoxic injury may be related to maintaining high HR for a long time, and decreasing the persistently high HR may be a reason why EP exerts a myocardial protective effect against exhaustive exercise-induced myocardial ischemic/hypoxic injury.

### EP exerted a myocardial protective effect by alleviating exhaustive exercise-induced myocardial ischemic/hypoxic injury

There are many ways to detect myocardial ischemic/hypoxic injury. cTnI is a blood biomarker with high specificity for detecting myocardial ischemic/hypoxic injury^22,35^. HBFP staining is a specific staining method for ischemia/hypoxia that is often used to detect early myocardial ischemic/hypoxic injury^36^. Normal cardiomyocytes are stained yellow by HBFP staining, but ischemic/hypoxic cardiomyocytes are stained scarlet^22^. In addition, the ST segment and T wave are ECG indexes that indicate myocardial ischemia/hypoxia; if this condition is present, the ST segment is obviously elevated or decreased, and the T wave is low, bidirectional or inverted^15^.

Shen et al.^37^ used HBFP staining to evaluate the degree of isoproterenol (ISO)-induced myocardial ischemic/hypoxic injury and found that there were large amounts of patchy dark red staining in ISO-induced ischemic/hypoxic cardiomyocytes, while the dark red-stained area were significantly decreased in EP + ISO cardiomyocytes. EP markedly attenuated ISO-induced myocardial ischemia/hypoxia. A study^35^ used HBFP staining of the myocardium revealed strong ischemia/hypoxia-related changes after high-intensity exercise, with a significantly higher myocardial ischemic/hypoxic density, but after high-intensity exercise following EP, the myocardial ischemic/hypoxic density was significantly reduced. Yuan et al.^38^ also found that EEP and LEP have protective effects on myocardial ischemic/hypoxic injury caused by exhaustive exercise used HBFP staining combined with serum cTnI levels.

As simple and noninvasive indexes for detecting myocardial ischemia/hypoxia, ST segment and T wave on ECG are widely used in sports medicine research. T wave changes induced by exercise stress tests can predict persistent ventricular tachycardia or ventricular fibrillation^39^, and they are related to cardiac death, particularly sudden cardiac death^40^. In response to myocardial ischemia, the ECG signal evolves into a towering T wave oscillation, which eventually leads to ventricular fibrillation^41^. Posa et al.^42^ occluded the left anterior descending branch of the coronary artery in rats that had undergone 6 weeks of voluntary wheel running; they found that the depression of the ST segment and the extent of myocardial ischemia/hypoxia were reduced in the exercised rats compared to the control group.

In this study, group EE had extensive patchy scarlet HBFP staining, a markedly elevated ST segment and an elevated T wave; no depression of the ST segment or inversion of the T wave was observed on ECG, but the plasma cTnI level was significantly elevated compared with that of group C. Some patchy scarlet staining appeared in group EEP + EE and group LEP + EE, but the degree of ischemia/hypoxia was significantly lower than that in group EE. The results of histological staining, ECG parameters, and plasma markers of myocardial injury all showed that the degree of myocardial ischemic/hypoxic injury caused by exhaustive exercise after EP was lower than that caused by exhaustive exercise without preparation. EP has a good protective effect against exhaustive exercise-induced myocardial ischemic/hypoxic injury. Moreover, ECG of exercise-induced myocardial ischemia/hypoxia showed ST segment and T wave elevation, which were distinct from the ST segment depression and T wave changes induced by other causes of myocardial ischemia/hypoxia^43,44^. The electrocardiographic characteristics of the ST segment and T wave after exhaustive exercise suggest that the site of exhaustive exercise-induced myocardial ischemic/hypoxic injury may be mainly concentrated in the subendocardial tissue^45^. EP can effectively reduce the HR after exhaustive exercise, thus prolonging the diastolic period of the heart, increasing the filling degree of peripheral blood vessels in the subendocardial myocardium, and reducing myocardial ischemic/hypoxic injury during exhaustive exercise. Several studies have shown that the protective effect of EP, which involves multiple endogenous triggers and recruits multiple signaling pathways, such as heat shock proteins^38^, mitoKATP channel opening^5,46^, PKCε^10^ and PKCδ^47^, were markedly upregulated.

ST segment and T wave reflect myocardial ischemia/hypoxia from the perspective of electrophysiology, while HBFP staining reflects the degree of myocardial ischemia/hypoxia from a histological perspective. As an index of ischemia/hypoxia, the correlation of ST segments and T waves with HBFP staining were investigated in the context of exhaustive exercise-induced myocardial ischemic/hypoxic injury. The results of the correlation analysis showed that the ST segment and T wave of the ECG were highly positively correlated with the ischemia/hypoxia area on the HBFP staining images. As the IHA on the HBFP staining images increased, the ST segment became elevated, and the T wave height gradually increased, which showed that histologically identified exhaustive exercise-induced myocardial ischemic/hypoxic injury was correlated with electrocardiographic characteristics. Thus, the ST segment and T wave can be used to evaluate exhaustive exercise-induced myocardial ischemia/hypoxia. These indexes enriched our findings on the myocardial protective effect of EP by providing direct measures of cardiac function.

### EP reduces the risk of malignant arrhythmias after exhaustive exercise

There is evidence that strenuous exercise, such as marathon running, increases the risk of sudden cardiac death^48^. Malignant arrhythmia is the main cause of exercise-induced sudden death^49,50^. A prolonged QT interval signified the risk of malignant arrhythmias and sudden cardiac death increased; however, the QT interval is easily affected by a variety of internal and external factors, such as electrolyte changes, exercise, and posture changes^15,51^. To eliminate the effect of HR on the QT interval, the Bazett formula for HR correction was used to calculate the QTc^52^.

A previous study found that the QTc interval of athletes was significantly longer shortly after a marathon than before and that this effect lasted for 24 h; additionally, long periods of strenuous exercise increased the risk index of arrhythmia^53^. Nie et al.^54^ also found that after brief high-intensity interval exercise (HIE), the QTc interval can be prolonged and that this effect can last for 24 h, thus increasing the associated risk. Chronic intermittent hypoxia exposure can prolong the QTc interval in rats, which can induce myocardial ischemia-related arrhythmias and sudden cardiac death^55^.

This study explored the ability of EP to reduce the risk of malignant arrhythmia after exhaustive exercise by analyzing the QTc interval on ECG in each group. The QTc interval was obviously prolonged after exhaustive exercise and was significantly shortened after exhaustive exercise with EP. Exhaustive exercise consists of a long period of strenuous exercise; the QTc interval of rat is prolonged after exhaustive exercise, indicating that exhaustive exercise may increase the risk of ventricular arrhythmia and sudden cardiac death in rat. However, when short-term, high-intensity intermittent aerobic exercise was performed 30 min and 24 h before exhaustive exercise, the QTc intervals were found to be significantly shorter in group EEP + EE and group LEP + EE than in group EE. It is speculated that EP may reduce the risk of arrhythmia after exhaustive exercise.

## Conclusions

EP has a protective effect against exhaustive exercise-induced myocardial ischemic/hypoxic injury by decreasing HR during exhaustive exercise, increasing myocardial blood supply, and reducing the incidence of malignant arrhythmia. The ECG characteristics of exhaustive exercise-induced myocardial ischemic/hypoxic injury were ST elevation and excessively tall T waves. The ST segment and T wave can also serve as indexes for evaluating exhaustive exercise-induced myocardial ischemia/hypoxia.

## Supplementary Information

Supplementary Information.

## Data Availability

The data that support the findings of this study are available from the corresponding author.
