# Supplementary material for: Exercise preconditioning improves electrocardiographic signs of myocardial ischemic/hypoxic injury and malignant arrhythmias occurring after exhaustive exercise in rats
Source: Sci Rep. 2022 Nov 5;12:18772. doi: 10.1038/s41598-022-23466-5 (PMC9637115; doi:10.1038/s41598-022-23466-5)
Supplement: Supplementary file 1 — Supplementary Information. [file 41598_2022_23466_MOESM1_ESM.docx]

Table 1 Raw data in heart rate of each rat (bpm)

| group  Number | group  C | group EEP | group LEP | group  EE | group E+E | group L+E |
| --- | --- | --- | --- | --- | --- | --- |
| 1 | 441 | 455 | 405 | 541 | 492 | 545 |
| 2 | 397 | 402 | 429 | 606 | 517 | 441 |
| 3 | 417 | 513 | 435 | 495 | 484 | 484 |
| 4 | 392 | 395 | 448 | 606 | 541 | 513 |
| 5 | 472 | 513 | 305 | 583 | 517 | 469 |
| 6 | 426 | 432 | 432 | 561 | 438 | 500 |
| 7 | 476 | 414 | 426 | 526 | 517 | 380 |
| 8 | 414 | 472 | 444 | 594 | 455 | 400 |
| 9 | 423 | 435 | 359 | 531 | 577 | 536 |
| 10 | 370 | 385 | 403 | 561 | 513 | 432 |
| 11 | 335 | 435 | 435 | 545 | 492 | 550 |
| 12 | 387 | 392 | 451 | 594 | 536 | 550 |
| 13 | 387 | 364 | 375 | 488 | 480 | 545 |
| 14 | 420 | 400 | 417 | 541 | 458 | 504 |
| 15 | 469 | 408 | 368 | 612 | 504 | 561 |
| 16 | 405 | 417 | 373 | 545 | 492 | 513 |
| 17 | 472 | 496 | 368 | 531 | 504 | 517 |
| 18 | 395 | 411 | 438 | 637 | 556 | 526 |
| 19 | 405 | 432 | 438 | 556 | 480 | 488 |
| 20 | 370 | 526 | 417 | 561 | 492 | 429 |

Table 2 Raw data in ST segment of each rat (1×100^-1^mV)

| group Number | group  C | group  EEP | group  LEP | group  EE | group  E+E | L+ group  E |
| --- | --- | --- | --- | --- | --- | --- |
| 1 | 6.28 | -4.33 | -0.75 | 2.85 | -2.85 | -1.2 |
| 2 | 4.13 | 0.8 | -2.25 | 2.72 | -3.06 | -10 |
| 3 | 4 | -0.67 | -1.5 | 0.54 | 3.43 | -8.5 |
| 4 | -5.42 | -0.55 | -1.3 | 4.78 | -2.86 | -8.38 |
| 5 | -3.28 | -0.29 | -2.33 | 6.1 | -4.09 | -8.23 |
| 6 | 2.06 | -3.88 | -0.28 | 13.78 | 2.67 | 1.46 |
| 7 | 3.33 | 1.09 | 1.5 | 10.18 | 0.8 | -6.06 |
| 8 | 6.33 | 1.13 | 0.87 | 10 | -0.31 | -4.61 |
| 9 | 0.55 | 2.23 | 2.54 | 15.38 | 3.21 | -4.56 |
| 10 | 0 | 2.12 | 4.14 | 16.4 | -2.87 | -10.17 |
| 11 | -0.55 | 1.78 | 3.8 | 19.4 | 7.67 | 0.33 |
| 12 | 5.33 | 3.13 | 3.7 | 18.81 | 10.83 | -7.1 |
| 13 | 4 | 5.64 | 4.18 | 24.13 | 6.8 | 4.68 |
| 14 | 6 | 8.78 | 4.88 | 29.81 | 4.31 | 11.75 |
| 15 | -1.55 | 8.75 | 4.68 | 26.16 | 5.55 | 10.56 |
| 16 | 5.11 | 7.11 | 4.7 | 35.63 | 13.33 | 8.92 |
| 17 | 6.55 | 9.82 | 4.5 | 41.96 | 12.25 | 15.73 |
| 18 | -6.75 | 12.56 | 5.17 | 39.8 | 16.56 | 30.4 |
| 19 | 4.18 | 12 | 6.22 | 36.12 | 21.11 | 18.64 |
| 20 | 5.11 | 12.63 | 9.73 | 43.58 | 19.27 | 36.47 |

Table 3 Raw data in T wave of each rat (1×100^-1^mV)

| group  Number | group  C | group  EEP | group  LEP | group  EE | group  E+E | group  L+E |
| --- | --- | --- | --- | --- | --- | --- |
| 1 | 22.57 | 12.8 | 12.8 | 19.86 | 16.27 | 2.23 |
| 2 | 23.6 | 17 | 13 | 21.04 | 18.5 | 20.6 |
| 3 | 14.8 | 18.22 | 16.22 | 21.93 | 17.273 | 17.4 |
| 4 | 23 | 18.67 | 18.6 | 24 | 18.1 | 14.18 |
| 5 | 21.57 | 18.87 | 16.9 | 24.25 | 26.559 | 18.75 |
| 6 | 20.875 | 20.6 | 18.45 | 26.26 | 19.33 | 20.83 |
| 7 | 16.22 | 20.73 | 20.89 | 28.12 | 24.1 | 20.84 |
| 8 | 16.33 | 21.36 | 19.375 | 30.38 | 25.571 | 20.92 |
| 9 | 19.44 | 25.82 | 20.57 | 32.9 | 26.79 | 21.36 |
| 10 | 14.25 | 24.56 | 20.8 | 31.69 | 18.07 | 22.66 |
| 11 | 20.286 | 23.11 | 16.71 | 31.47 | 30.067 | 23 |
| 12 | 25.857 | 23.78 | 23.25 | 35.62 | 28.508 | 24.64 |
| 13 | 19.5 | 24.55 | 21.62 | 36 | 33 | 26 |
| 14 | 22.75 | 22.89 | 26.57 | 36.1 | 28.692 | 26.55 |
| 15 | 12.33 | 24.87 | 23.08 | 38.95 | 28.875 | 26.67 |
| 16 | 22.11 | 21.91 | 21 | 42.35 | 28.278 | 35.18 |
| 17 | 16.33 | 27.38 | 23.37 | 46.33 | 31.438 | 27.7 |
| 18 | 19.75 | 39.77 | 24.1 | 55.89 | 28.67 | 28.11 |
| 19 | 19.45 | 28.75 | 22.5 | 65.62 | 33.667 | 27.62 |
| 20 | 24 | 37.75 | 29.55 | 75.52 | 43.2 | 42.33 |

Table 4 Raw data in QTc interval of each rat (ms)

| group  Number | group  C | group  EEP | group  LEP | group  EE | group  E+E | group  L+E |
| --- | --- | --- | --- | --- | --- | --- |
| 1 | 181.9 | 173.45 | 186.1 | 196.67 | 185.73 | 197.96 |
| 2 | 164.9 | 181.82 | 169.42 | 190.1 | 163.72 | 198.22 |
| 3 | 151.9 | 189.89 | 161.11 | 201 | 167.79 | 179.75 |
| 4 | 171.94 | 173.64 | 177.82 | 195.22 | 187.09 | 184.56 |
| 5 | 178.93 | 149.88 | 181.94 | 198.63 | 192.07 | 169.56 |
| 6 | 181.13 | 183.1 | 176.67 | 205.62 | 186.46 | 180.72 |
| 7 | 181.33 | 146.77 | 188.33 | 204.62 | 176.28 | 180.45 |
| 8 | 164.78 | 182.29 | 170.3 | 191.78 | 185.38 | 168.62 |
| 9 | 180.78 | 169.11 | 162.7 | 201.92 | 191.65 | 179.62 |
| 10 | 167.67 | 168.56 | 188.5 | 198.92 | 191.27 | 186.5 |
| 11 | 140.89 | 182.88 | 179.13 | 187 | 185.24 | 186.72 |
| 12 | 154.56 | 185 | 162.71 | 202.37 | 194.62 | 193.62 |
| 13 | 166.13 | 183.22 | 171.29 | 207.24 | 173.88 | 193.45 |
| 14 | 186 | 194.33 | 184.8 | 185.6 | 175.32 | 175.63 |
| 15 | 168 | 175.27 | 188.22 | 202.56 | 197.75 | 191.87 |
| 16 | 167.67 | 194.75 | 164 | 194.36 | 196.07 | 170.13 |
| 17 | 171 | 184 | 166.32 | 186.1 | 163.11 | 169.29 |
| 18 | 184.86 | 190.38 | 195 | 186.33 | 183.56 | 175.2 |
| 19 | 184.71 | 186.88 | 172.5 | 196 | 205 | 154.31 |
| 20 | 165.44 | 188.38 | 172.38 | 194.13 | 162.1 | 198.55 |
